# Neuronal precursor cells with dopaminergic commitment in the rostral migratory stream of the mouse

**DOI:** 10.1038/s41598-019-49920-5

**Published:** 2019-09-16

**Authors:** Kerstin Schweyer, Corinna Rüschoff-Steiner, Oscar Arias-Carrión, Wolfgang H. Oertel, Thomas W. Rösler, Günter U. Höglinger

**Affiliations:** 1 Department of Neurology, Technical University of Munich, School of Medicine, Munich, Germany; 2 Department of Translational Neurodegeneration, German Center for Neurodegenerative Diseases, Munich, Germany; 3 Department of Neurology, University of Marburg, Marburg, Germany; 4 Department of Neonatology, University Hospital Zurich, University of Zurich, Zurich, Switzerland; 5 Unit of Movement and Sleep Disorders, Hospital General Dr. Manuel Gea González, Mexico City, Mexico; 6 Department of Neurology, Hannover Medical School, Hannover, Germany

**Keywords:** Parkinson's disease, Parkinson's disease

## Abstract

Neuroblasts born in the subventricular zone of adult mammals migrate via the rostral migratory stream into the granular cell layer or periglomerular layer of the olfactory bulb to differentiate into interneurons. To analyze if new neurons in the granular cell layer or periglomerular layer have different origins, we inserted a physical barrier into the rostral migratory stream, depleted cell proliferation with cytarabine infusions, labeled newborn cells with bromodeoxyuridine, and sacrificed mice after short-term (0, 2, or 14 days) or long-term (55 or 105 days) intervals. After short-term survival, the subventricular zone and rostral migratory stream rapidly repopulated with bromodeoxyuridine^+^ cells after cytarabine-induced depletion. Nestin, glial fibrillary acidic protein and the PAX6 were expressed in bromodeoxyuridine^+^ cells within the rostral migratory stream downstream of the physical barrier. After long-term survival after physical barrier implantation, bromodeoxyuridine^+^ neurons were significantly reduced in the granular cell layer, but bromodeoxyuridine^+^ and dopaminergic neurons in the periglomerular layer remained unaffected by the physical barrier. Thus, newborn neurons for the granular cell layer are mainly recruited from neural stem cells located in the subventricular zone, but new neurons for the periglomerular layer with dopaminergic predisposition can rise as well from neuronal stem or precursor cells in the rostral migratory stream.

## Introduction

Neurogenesis is continuously ongoing in the adult mammalian brain. There are two known neurogenic regions, the subventricular zone (SVZ) of the ventral telencephalon underlying the ependymal layer of the lateral ventricles^1,2^ and the subgranular zone of the dentate gyrus in the hippocampus^3^. In other brain areas, for example the striatum or the neocortex, the existence of spontaneous or lesion-induced neurogenesis is still under debate^4,5^.

Specific astrocytes of the SVZ, which are derived from neonatal radial glia^6^, act as adult neural stem cells (NSCs, so-called type B cells)^7^. Type B cells give birth to rapidly dividing, transit-amplifying precursor cells (type C cells), which give rise to migrating neuroblasts (type A cells)^8^. These neuroblasts migrate by an interconnected pattern, termed chain migration, via the rostral migratory stream (RMS) to the olfactory bulb (OB). During their migration, type A cells continuously divide and finally differentiate into interneurons^9,10^. After arriving at the OB, approximately 90% of the neuroblasts migrate radially to the granule cell layer (GCL) and approximately 10% to the periglomerular layer (PGL)^11^. Numerous investigations have been carried out on the functional anatomy of the SVZ-RMS-OB system. Initially, the RMS has been mostly considered as a mere connecting pathway between SVZ and OB. Thus, the existence of adult NSCs and neural precursor cells (NPCs) located within the RMS, with the potential to create new interneurons for the OB, has been disregarded for a long time. During the last decade, the potential of the RMS to act as a neurogenic niche by itself has been discovered and several studies on stem cells in the RMS has been conducted^12–15^.

As considered functionally relevant^16^, newborn dopaminergic neurons in the PGL of the OB are particularly interesting. The anatomical localization of adult NSCs and NPCs with the potential to produce these dopaminergic neurons in the adult PGL remains disputed. Some researchers consider them to be localized in the dorsal SVZ^17^, but others in the RMS^18,19^.

Previous studies addressed this question by retroviral labeling of cells derived from the SVZ or RMS, respectively^19^, or by studying the SVZ-RMS system after depleting it completely from proliferating cells by infusion of the antimitotic drug cytarabine (AraC)^13,15^. Also, a method to block neuroblast migration in the proximal RMS by insertion of a physical barrier (PB) has been reported^15^. These studies suggested that the RMS might produce dopaminergic cells independently from the SVZ. However, previous work did not unequivocally resolve the question of the origin of the dopaminergic cells in the PGL, since technical limitations did not fully rule out the possibility that neuroblasts immigrating from the SVZ into the RMS might be their origin.

In the present study, we combined these methodological approaches in an attempt to resolve the question about the capacity of the RMS to generate dopaminergic cells for the PGL. First, we implanted a PB into the upstream (caudal) RMS to block the immigration of neuroblasts from the SVZ. Then, we depleted the SVZ from proliferating cells and migrating neuroblasts by AraC infusion. When NSC and NPC proliferation in the SVZ and RMS resumed after termination of the AraC infusion, we labeled them with the thymidine analog bromodeoxyuridine (BrdU). This approach allowed us to conclude that neuroblasts arriving at the OB were originating from the RMS (in the presence of a PB), or from either RMS or SVZ (in the absence of a PB). Our results indicate that NSCs and NPCs located in the RMS have indeed the potential to generate dopaminergic neurons for the OB.

## Results

### Depletion and repopulation of the SVZ and RMS with proliferating precursor cells after AraC treatment

**Figure 1:**
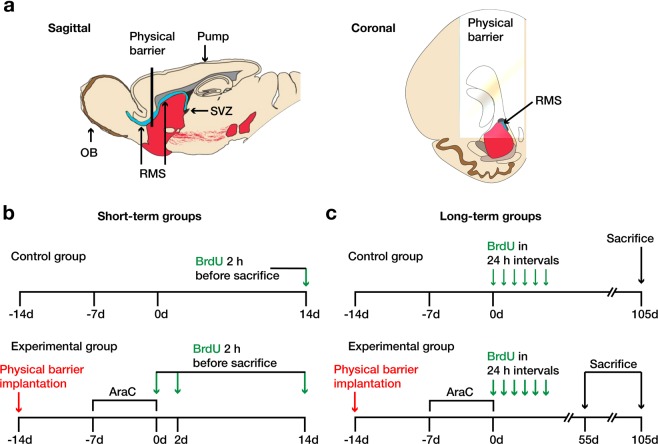
Overview of experimental procedures. (**a**) Sagittal and coronal view of the rostral migratory stream (RMS) illustrating the position of the physical barrier (PB) and the osmotic minipump catheter tip. The PB was implanted +1.7 mm rostral to bregma, interrupting migration of neuroblasts from the right subventricular zone (SVZ) to the olfactory bulb (OB). A catheter connected to an osmotic minipump was implanted on the brain surface +1.1 mm rostral to bregma to infuse AraC on the brain surface in proximity to the SVZ. Schematic illustration of experimental treatments in the groups of mice sacrificed at short-term (**b**) or long-term (**c**) intervals after start of the experimentation (i.e. day 0). In the short-term groups, mice were sacrificed at 0, 2, or 14 days after start of the experimentation, 2 hours after intraperitoneal bromodeoxyuridine (BrdU) injection, to study precursor cell proliferation. In the long-term groups, mice were injected with BrdU 6 times in 24-hour intervals after start of the experimentation to label proliferating precursor cells and sacrificed at 55 or 105 days, to study their migration and phenotypic differentiation.

To investigate the antimitotic effect of AraC on NSC and NPC proliferation, the SVZ and RMS were immunohistochemically stained for BrdU, incorporated into the DNA of cells which had undergone mitosis within the 2 h before sacrifice (Fig. 1b). In both regions, BrdU^+^ cells were counted in the control group to study the normal rate of NSC/NPC proliferation, and in the three short-term experimental groups, i.e. 0, 2 and 14 days after AraC infusion, to study the depletion and repopulation of the SVZ and RMS with neuroblasts (Fig. 1).

**Figure 2:**
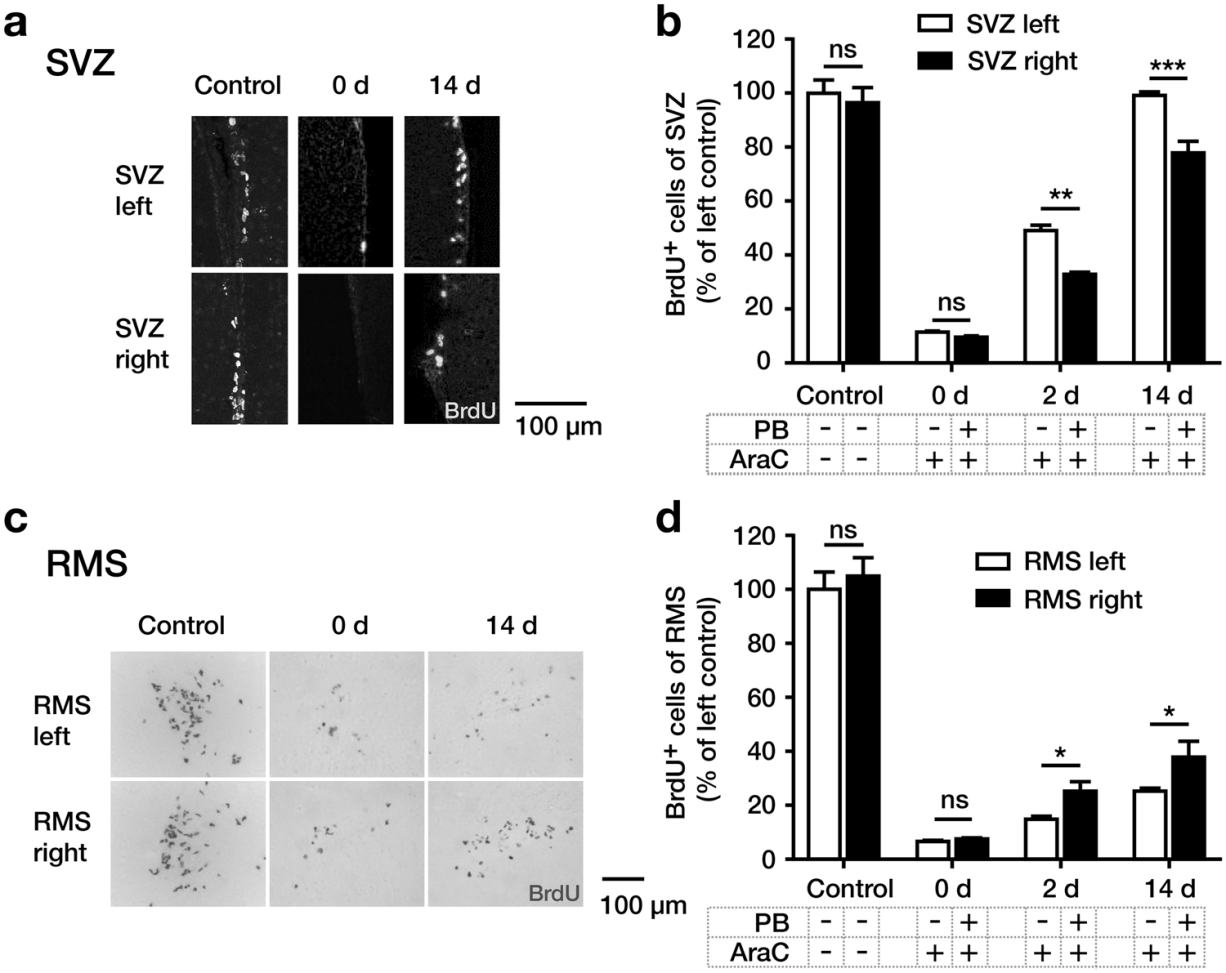
Depletion and repopulation of the subventricular zone (SVZ) and rostral migratory stream (RMS) with proliferating neural stem and precursor cells after AraC treatment. (**a**) Representative coronal sections of the SVZ immunostained for BrdU, showing almost complete depletion on day 0 and strong recovery on day 14 after AraC treatment on both sides, with lower cell numbers in the right SVZ (upstream (caudal) of the physical barrier (PB)) vs. the left SVZ (without PB). (**b**) Quantification of BrdU^+^ cells in the right and left SVZ of the control group (without AraC and PB) and on day 0, 2 and 14 after AraC infusions (left control side defined 100% values). BrdU^+^ cells were counted in the SVZ which was defined by the coordinates +0,002 mm to +0,0845 mm relative to bregma using the Paxinos mouse atlas (**c**) Representative coronal sections of the RMS (~2,0 mm rostral to bregma) immunostained for BrdU, showing almost complete depletion on day 0 and incomplete recovery on day 14 after AraC treatment on both sides, with higher cell numbers in the right RMS (downstream (rostral) of the PB) vs. the left RMS (without PB). (**d**) Quantification of BrdU^+^ cells in the RMS of the control group (left side defines 100% values) and on day 0, 2 and 14 after AraC-infusion. BrdU^+^ cells were counted in the RMS which was defined by the coordinates +1,85 mm to +2,04 mm relative to bregma using the Paxinos mouse atlas. Data were analyzed by using two-way ANOVA with Fisher’s LSD post-hoc test and are shown as mean + SEM. A p-value < 0.05 was considered as statistically significant: *p < 0.05; **p < 0.01; ***p < 0.001, ns = not significant.

In the SVZ, mice showed on day 0 after AraC infusion a strong reduction of BrdU^+^ proliferating NSCs/NPCs on both the right side (with PB), and the left side (without PB), compared to control mice without AraC infusion and without PB. On day 2, a partial recovery of proliferation in the SVZ was observed, with markedly more BrdU^+^ cells on the left side without the barrier compared to the right side. On day 14, the number of BrdU^+^ cells in the left SVZ reached the number of untreated control mice again, while the right SVZ significantly lagged behind (Fig. 2a,b).

In the RMS at day 0 after AraC infusion, there was an almost complete loss of BrdU^+^ neural stem and precursor cells on both sides. Similar to the SVZ, BrdU^+^ cells repopulated the RMS, albeit only partially, on both sides during the observed time course. Contrary to the SVZ, the number of BrdU^+^ cells in the RMS was significantly higher on the right side (with PB) compared to the left side (without PB), both on day 2 and 14 (Fig. 2c,d).

These data suggest that the RMS downstream (rostral) of the PB activated local cell proliferation upon blockade of immigration of SVZ-derived precursor cells. To define these newborn cells from the RMS further, we studied if BrdU^+^ cells in the RMS would express markers characteristic of stem and precursor cells.

### Expression of stem and precursor cell markers in the RMS

To characterize the cells, which could give rise to the BrdU^+^ cells in the RMS rostral of the PB, we performed co-immunofluorescence stainings with markers for NSCs (Nestin and GFAP) and NPCs (PSA-NCAM, Pax6)^8^.

**Figure 3:**
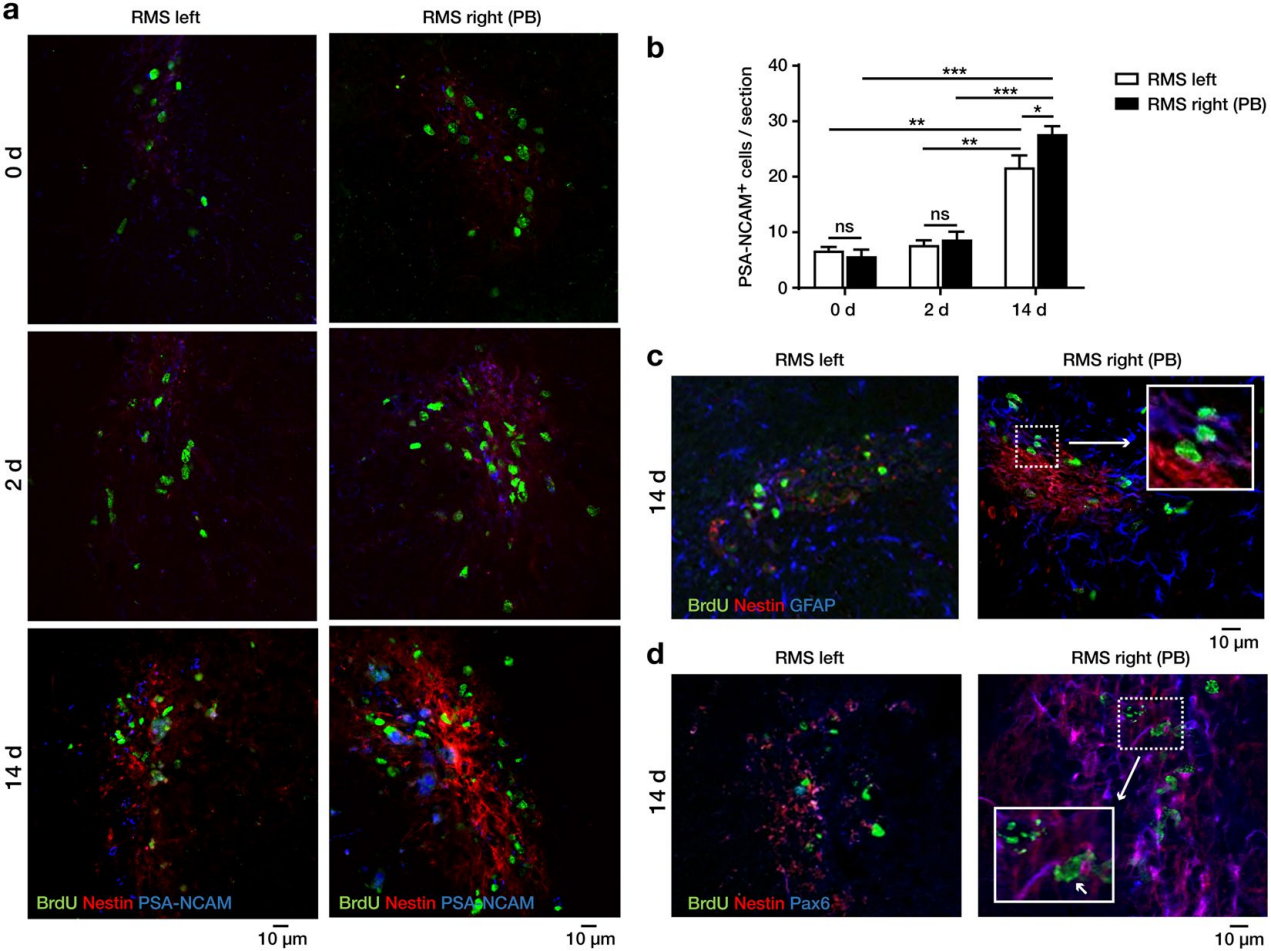
Stem cell marker expression in the rostral migratory stream (RMS). Immunofluorescent staining was performed on coronal sections in the RMS on the right side (downstream (rostral) of the physical barrier (PB)) and the left side (without PB) at defined time-points after AraC infusion. (**a**) Stainings for Nestin (labeling neural stem and precursor cells), PSA-NCAM (labeling neuronal precursor cells) and BrdU (labeling proliferating cells), showed a gradual increase in Nestin and BrdU and a slight increase of PSA-NCAM immunoreactivity from day 0 to day 14, with higher levels on the right side (with PB) compared to the left side (without PB). (**b**) Quantification of PSA-NCAM^+^ cells/section in the right and left RMS on day 0, 2 and 14 after AraC infusions. (**c**) Coronal sections of the RMS stained for BrdU, Nestin and GFAP (labeling neural stem and progenitor cells), coronal section of the right RMS (with PB) displays several GFAP^+^ Nestin^+^ cytoplasmic structures in immediate contact to BrdU^+^ nuclei (arrow), which were virtually absent in the left RMS, suggesting the presence of activated resident Nestin^+^ and GFAP^+^ stem and precursor cells in the RMS downstream of the PB. (**d**) Coronal sections of the RMS stained for BrdU, Nestin and Pax6 (a marker for neural stem and progenitor cells and a dopaminergic fate determining factor), coronal section of the right RMS (with PB) exhibits several Pax6^+^ and Nestin^+^ cytoplasmic structures in immediate contact to BrdU^+^ nuclei (arrow), which were virtually absent in the left RMS, suggesting that reactivated stem cells in the RMS might favour a dopaminergic fate determination. Data were analyzed by using two-way ANOVA with Sidak’s multiple comparison test and are shown as mean + SEM. A p-value < 0.05 was considered as statistically significant: *p < 0.05; **p < 0.01; ***p < 0.001; ns = not significant.

On day 2 and 14 (Fig. 3a), increased expression of Nestin was detected in the right vs. the left RMS with and without the PB, respectively. Nestin expression as a consequence of physical traumatization during PB implantation was considered unlikely because of its delayed temporal expression and its localization distant from the PB.

Expectedly, we found PSA-NCAM^+^ and Nestin^-^ neuronal precursor cells to follow the time course of BrdU^+^ cells, showing a gradual increase from day 0 to day 14, with higher levels on the right side (downstream of the PB) compared to the left side (without PB) (Fig. 3a,b).

Next, we stained the RMS for GFAP, a marker for astrocytes and a subpopulation of type B NSCs in the SVZ^8^, and for Pax6, a transcription factor promoting dopaminergic neurogenesis in type A and C NPCs^19–21^. These stainings were carefully investigated by confocal microscopy. Unequivocal co-localization of Nestin^+^ and GFAP^+^ cytoplasmic structures immediately adjacent to BrdU^+^ nuclei were found in the right RMS (downstream of the PB) (Fig. 3c), whereas such cells were virtually absent on the left side (without PB), suggesting a reactivation of NSCs and NPCs in the RMS. Pax6^+^/Nestin^+^ cytoplasmic structures in close vicinity to BrdU^+^ nuclei were found in the right RMS (downstream of the PB) (Fig. 3d), but were absent on the left side (without PB).

Together, these data suggest that the RMS downstream of the PB activated resident Nestin^+^ GFAP^+^ stem and precursor cells, which co-express Pax6, suggesting that they might favour a dopaminergic phenotype. Therefore, we analyzed the long-term differentiation of new-born neuroblasts.

### Long-term effects of PB implantation on BrdU^+^ cells in the OB

In animals subjected to long-term survival, a PB was implanted on the right RMS (Fig. 1a) to prevent the immigration of SVZ-derived neuroblasts into the RMS. The left side contained no PB. Animals were infused with AraC to deplete neurogenesis and then injected with BrdU daily for 6 days thereafter to label cells born during that period. At 55 and 105 days after AraC infusion, the terminal migration and phenotypic differentiation pattern of the new-born BrdU^+^ precursor cells were analyzed in the OB.

**Figure 4:**
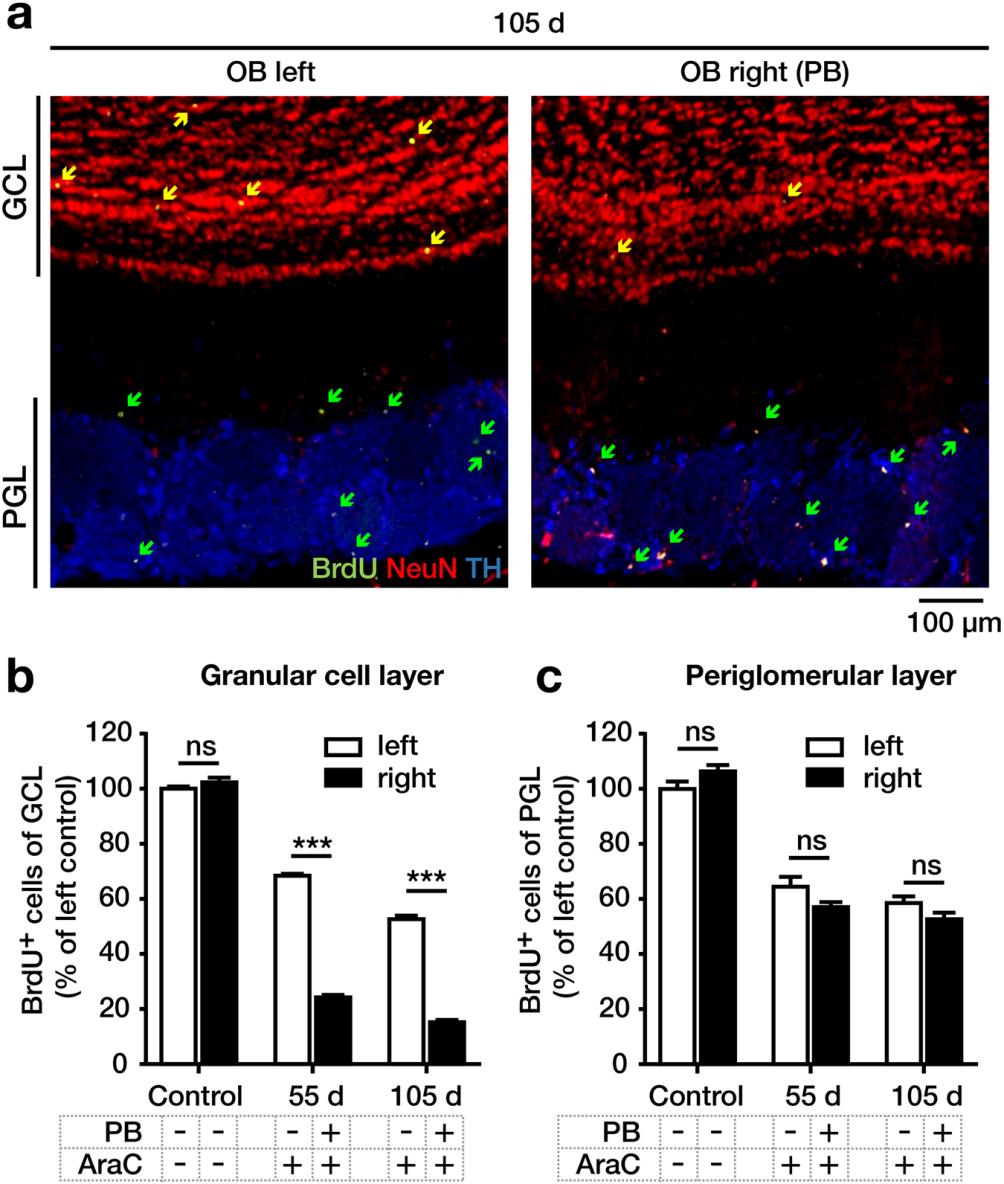
Neurogenesis in the olfactory bulb (OB). Newborn cells were analyzed in the granular cell layer (GCL) and the periglomerular layer (PGL) without AraC infusion (control) or at 55 or 105 days after AraC infusion on the left side (no physical barrier (PB)) and right side (with PB). (**a**) Coronal sections of the left and right OB on day 105 stained for BrdU (green), NeuN (red) and TH (blue). The GCL corresponds to the red cell layers on top. The PGL corresponds to the blue cell clusters on the bottom. Note the reduction in the number of BrdU^+^ cells (arrows) in the GCL of the right vs. the left side, which is not observed in the PGL. (**b,c**) Numbers of BrdU^+^ cells in controls and on day 55 and 105 after AraC infusion, showing a significant effect of the PB in the GCL (**b**), but not in the PGL (**c**). The left side of the control group was set to 100%. BrdU^+^ cells were quantified in the PGL and GCL in the OB (3,74 mm to 6,86 mm relative to bregma). Data were analyzed by using two-way ANOVA with Fisher’s LSD post-hoc test and are shown as mean + SEM. A p-value < 0.05 was considered as statistically significant: ***p < 0.001, ns = not significant.

In the GCL of the OB, the number of BrdU^+^ cells was significantly reduced at 55 and 105 days after AraC infusion compared to the control group without AraC infusion, reflecting the transient reduction in neural stem and precursor cell generation. The number of BrdU^+^ cells in the GCL was significantly lower on the right side (downstream (rostral) of the PB) compared to the left side (without PB), reflecting the blocked migration of precursor cells from the SVZ via the RMS into the GCL (Fig. 4a,b).

Also, in the PGL of the OB, the number of BrdU^+^ cells was reduced at 55 and 105 days after AraC infusion compared to the control group without AraC infusion, reflecting the transient reduction in precursor cell generation. However, no significant differences in the numbers of BrdU^+^ cells were detected by comparing the right vs. the left side (Fig. 4a,c). This finding demonstrated that the PB did not impair the immigration of neuroblasts into the PGL, suggesting that in our experiment they were born downstream of the PB.

### Turnover of dopaminergic neurons in the PGL

Since the PGL comprises dopaminergic neurons, we study in detail their turnover after PB implantation.

**Figure 5:**
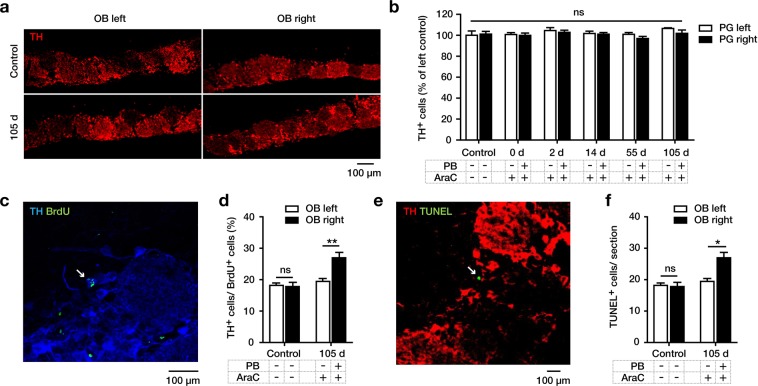
Turnover of dopaminergic cells in the periglomerular layer (PGL) of the olfactory bulb (OB). Analyses were done on the left side (without physical barrier (PB)) and right side (with PB) in animals without AraC infusion (control) or at 105 days after AraC infusion. (**a**) The PGL of the left and right OB was immunostained for tyrosine hydroxylase (TH) to identify dopaminergic cells on day 105 after the start of BrdU labeling. (**b**) Numbers of TH^+^ cells in the PGL on both sides were not different between all experimental groups. The left side of the long-term control group was set to 100%. (**c**) PGL of the right OB of the experimental group on day 105 showing a representative BrdU^+^/TH^+^-cell. (**d**) Percentage of TH^+^-cells among BrdU^+^ in the PGL of the left and right OB in animals without intervention (controls) or treated with AraC and with (right side) or without PB (left side) implantation, showing an increased TH^+^/BrdU^+^-percentage after PB implantation, indicating a higher rate of dopaminergic neurogenesis downstream of the PB. (**e**) PGL, as delineated by TH immunostaining, in the right OB on day 105 showing a representative TUNEL^+^ cell. (**f**) Average number of TUNEL^+^ cells per section in the PGL of the left and right OB in animals without intervention (controls) or treated with AraC and with (right side) or without (left side) PB implantation, showing an increased number of TUNEL^+^ cells after PB-implantation indicating a higher rate of apoptosis downstream of the PB. Data were analyzed by using two-way ANOVA with Fisher’s LSD post-hoc test and are shown as mean + SEM. A p-value < 0.05 was considered as statistically significant: *p < 0.05; **p < 0.01; ns = not significant.

First, we quantified the total numbers of dopaminergic (TH^+^) neurons over the whole timeframe of our experiments (all short- and long-term groups). We observed a stable number of dopaminergic cells across all groups (Fig. 5a,b), demonstrating that our experimental interventions did not significantly affect their total number.

Then, we quantified in the PGL of controls and AraC-treated animals of the long-term groups (105 days of survival) the proportion of BrdU^+^ neurons which co-immunolabeled for TH (i.e. dopaminergic cells among all new-born cells). Interestingly, AraC-treatment without PB did not change the TH^+^ among the BrdU^+^ cells, but additional PB implantation significantly increased this proportion (Fig. 5c,d).

Finally, we quantified the number of TUNEL^+^ apoptotic cells within the PGL, as delineated by TH immunostaining. Again, AraC treatment without PB did not change the number of TUNEL^+^ cells, but additional PB implantation significantly increased this number (Fig. 5e,f).

Together, these data suggest that PB implantation increased the cell turnover (i.e. birth and death rates) in the PGL of the OB, but did not alter the absolute numbers of TH^+^ neurons therein.

## Discussion

The SVZ-RMS-OB system is known to be one of two regions where new neurons are continuously generated in adult mammalian brains. After the asymmetric division of NSCs (type B cells) or transit amplifying NPCs (type C cells) in the SVZ, neuroblasts (type A cells) migrate via the RMS to the OB and integrate as interneurons. Among the newly generated neurons in the PGL of the OB, a proportion of about 20% shows a dopaminergic phenotype. The aim of the present study was to investigate the capacity of the of NSCs or early NPCs in the RMS to generate these dopaminergic neurons independently from the SVZ. Therefore, we created a mouse model where the right RMS was mechanically interrupted by a PB. Neural precursor cells were depleted by AraC infusion and newly proliferating NPCs were labeled by BrdU application. The experimental groups were followed up for short-term (0, 2 and 14 days) or long-term (55 and 105 days) observational periods prior to histological analyses. Animals in the control group received no PB implantation and no AraC treatment.

We aimed to investigate if the RMS downstream of the PB implantation has the potential to act as a neurogenic niche. Previous experimental studies provided consistent results indicating the RMS to be a source for NSCs which can give rise to granular and periglomerular interneurons^12,17–19,22^. Nevertheless, the methodological approach in these experiments did not allow to conclude with certainty about the anatomical location of the NSCs which are able to generate dopaminergic neurons in the PGL. We combined physical interruption of cell migration with antimitotic treatment to deplete the RMS from migrating neuroblasts, and pulse-labeled newborn neuroblasts with BrdU in defined time intervals, to analyze first the proliferative response in the RMS downstream of the PB in short-term intervals after AraC infusion, and secondly the survival and terminal differentiation of newborn neurons in the OB downstream of the PB in long-term intervals after AraC infusion. This complex experimental setting allowed us to demonstrate that the RMS downstream of the PB can activate a stem cell niche upon blockade of migration of SVZ-derived neuroblasts, and secondly that dopaminergic neurons in the PGL can indeed be born in the rostral part of the RMS.

First, the short-term experiments demonstrated that AraC treatment effectively and comprehensively eliminated precursor cell proliferation in the RMS and SVZ and therefore represents an appropriate method to study stem cell proliferation in the RMS-SVZ-system in a temporally controlled manner. We observed cell proliferation in the SVZ after AraC treatment and found a lower number of BrdU^+^ cells in the SVZ caudal to the interrupted RMS. Although our findings of immediately reduced proliferation in the SVZ after interruption of the ipsilateral RMS are in line with previous studies^23^, exact mechanisms remain unclear. As olfactory input was shown to affect proliferation in the SVZ, decreased proliferation after rostral injury may reflect unilaterally reduced olfactory stimulation^23,24^^.^ Furthermore, we investigated the cell proliferation in the RMS and found higher numbers of BrdU^+^ cells in the right RMS downstream of the PB compared to the left side without PB. This contrasts the results of a prior similar experiment in rats without AraC infusion (however with PB implantation), reporting equal numbers of BrdU^+^ cells on both sides^15^. Since we excluded migration from the SVZ with the PB and depleted rapidly dividing cells in the RMS with AraC, we assume that proliferation in this area starts from quiescent NPCs residing in the RMS. The significant increase in the number of BrdU^+^ cells downstream of the PB as opposed to the side without PB suggests that this niche contains NSCs which appear to increase their proliferative capacity upon reduced immigration of neuroblasts from the SVZ. It cannot fully be excluded that the higher number of BrdU^+^ cells reflects a reactive proliferation of glia cells following the surgical implantation of the PB. However, since reactive gliosis appears immediately after brain damage and declines with time^25^, this possibility seems rather unlikely to be the case in our experiments, where the proliferative capacity increased over time.

We then aimed to characterize the cells in this niche more closely using established stem and precursor cell markers. Previous studies^26,27^ suggested that repopulation of the SVZ after the antimitotic AraC treatment starts from Nestin^+^ GFAP^+^ type B cells, producing transit-amplifying C cells, which then create new PSA-NCAM^+^ neuroblasts to migrate to the OB. Our observations show that - similar to the SVZ – the niche in the RMS also contains Nestin^+^ GFAP^+^ cells which appear to incorporate BrdU immediately after termination of the AraC treatment. Interestingly, Nestin expression increased with time after termination of the AraC infusion on the side of PB implantation, but not on the side without PB. This suggests reactivation of this neurogenic niche after blocking the immigration of SVZ-derived neuroblasts. Demonstration of Nestin^+^/GFAP^+^/BrdU^+^ cells in the RMS strongly suggests that these cells are slowly dividing neuronal precursor cells, since a combination of markers was previously only reported on type B cells in the SVZ^7,12^. The Pax6 immunoreactivity of the cells resident in this nice indicates that the reactivated precursor cells in the RMS favour a dopaminergic fate determination of their descendant precursor cells^19,20^, although the final phenotype cannot be predicted with certainty in our experiment.

Secondly, the long-term experiments demonstrated that interruption of migration of SVZ-derived neuroblasts from the SVZ into the RMS significantly reduced the number of newborn neurons in the GCL but not in the PGL. This provides further evidence that newborn periglomerular cells can have their origin in the RMS whereas granular neurons mainly rise from NSCs located in the SVZ. NSCs located in the RMS were shown to provide primarily (specifically dopaminergic) interneurons for the PGL. These results support our hypothesis that the RMS represents a neurogenic niche on its own, which is in line with prior studies^15,19,22^.

Specifically, our findings support the idea of a regionalized specialization of NSCs distributed within the SVZ-RMS-OB system. Different regions in the SVZ-RMS-OB system appear to produce distinct subgroups of interneurons for the OB. This concept has been proposed in previous studies^17,19,20^. Particularly Merkle and colleagues confirmed regionalization by labeling 15 different NSC regions of the SVZ and RMS with a Cre recombinase-expressing adenovirus^17^. Going beyond prior knowledge, we aimed to gain specifically insight into the neurogenesis of dopaminergic interneurons in the PGL. We demonstrated a significant increase in dopaminergic (TH^+^) neurons among the newborn (BrdU^+^) cells on the PB-interrupted side, suggesting that cells born by the reactivated NPCs in the RMS differentiate more likely into dopaminergic cells, as compared to newborn neurons with predominant origin from the SVZ on the contralateral hemisphere without a PB. It has to be considered that under physiological conditions TH^+^ cells were shown to derive both from the SVZ and the RMS^19^. Thus, experimental interruption of the RMS provoked a change in the activity of more rostral localized NPCs.

Despite the increased number of newborn TH^+^ cells, we did not find any significant changes in the total number of TH^+^ cells in the PGL upon PB implantation. This prompted us to investigate apoptotic cells in the right OB using a TUNEL staining. Indeed, an increased rate of apoptosis occurred, supporting the hypothesis of an intrinsic regulation of dopaminergic cell turnover in the OB maintaining the number of dopaminergic cells at a constant level. Consistently, prior work suggested the existence of an intrinsic signal pathway adapting cell proliferation in the RMS to the demand of periglomerular interneurons in the OB^9,28,29^.

In conclusion, we provide evidence that the RMS works as a niche that favours differentiation into a dopaminergic cell type. It harbors adult NSCs and has an important role in regulating of PGL cell number and the dopaminergic cell fate. As suggested in prior work^29^, the RMS is indeed able to adapt to and compensate specific conditions, such as reduced migration of SVZ-derived neuroblasts, by enhancing its proliferative activity.

## Methods

### Animals

Male wild-type C57BL/6 mice (Charles River, Sulzfeld, Germany) 10 weeks of age with a weight of 20 to 25 g were used. They were housed in standard cages with *ad libitum* access to food and water. The room temperature was 23 °C with a 12-hour light-dark cycle. Animal experiments were performed according to German legislation and the EU Council Directive 86/609/EE. Experiments were approved by the Regierungspräsidium Gießen (V54-19c20-15(1)MR20/15Nr.41/2009). Mice were randomized into the experimental groups.

### Implantation of the PB

PBs were implanted in mice as described previously for rats^15^. Mice were anesthetized by a mixture of ketamine (80 mg/kg) and xylazine (4 mg/kg). The cranium was opened carefully by a 0.5 cm midsagittal skin incision. A small hole was drilled in the skull (anterior-posterior +1.7 mm, mediolateral +2.0 mm; stereotactic coordinates corresponding to the mouse brain atlas^30^). The PB, a sterile polypropylene sheet (width 2.0 mm, length 3.5 mm), was implanted unilaterally into the right RMS 1.7 mm rostral to bregma and 3.5 mm ventral from the dura mater (Fig. 1a). After surgery, the skin was sutured.

### AraC treatment

Seven days after PB implantation, an osmotic minipump (Alzet Osmotic Pumps, Brain Infusion Kit 3, Cupertino, CA) was implanted in animals of the experimental groups (Fig. 1) to administer an AraC solution (2% AraC in sterile saline). Surgery procedures were similar to PB implantation, preparing a bur hole trepanation at stereotactic coordinates relative to bregma (anterior-posterior 0 mm, mediolateral +1.1 mm, dorsoventral −1.0 mm)^30^. Cannulas of 1 mm length were fixed onto the surface of the brain and connected subcutaneously to the pump implanted between the scapulae. AraC was delivered at a flow rate of 0.5 μl/h for 7 d.

### BrdU administration

Animals were subjected to different protocols of intraperitoneal BrdU (100 mg/kg) injection. One short-term control group (without PB and AraC infusion) was sacrificed 2 h and 14 d after a single BrdU injection, to study the normal rate of precursor cell proliferation. In the short-term experimental groups, mice were sacrificed at 0, 2, or 14 days after the end of the AraC infusion, 2 h after a single BrdU injection, to verify the effective blocking of precursor cell proliferation and its re-emergence (Fig. 1b).

The long-term control mice received 6 BrdU injections in 24 h intervals and were sacrificed on day 105 after the first injection, to study the normal migration and differentiation pattern of neural precursor cells in the OB (Fig. 1c). Mice of the long-term experimental groups received 6 BrdU injections in 24 h intervals, starting after the end of AraC infusion, and were sacrificed on day 55 or 105, to study the migration and differentiation pattern of neural precursor cells in the OB from stem cells downstream of the PB.

### Tissue sample collection and processing

Mice were sacrificed by intraperitoneal injection of pentobarbital and then intracardially perfused with ice-cooled 0.9% NaCl solution for 3 min followed by 4% paraformaldehyde (PFA) in 0.1 M phosphate buffer (PBS). After decapitation, brains were removed and post-fixed in 4% PFA at 4 °C for 2 days. Afterwards brains were cryoprotected in 30% sucrose solution at −20 °C, cut into sections of 30 µm thickness on a cryomicrotome, collected in 10 regularly-spaced series and stored in antifreeze solution containing 30% ethylene glycol and 30% glycerine at −20 °C.

### DAB immunostaining

3,3′-diaminobenzidine (DAB) staining was performed for BrdU and tyrosine hydroxylase (TH). Free-floating sections were incubated for 15 min in blocking solution consisting of 0.1 M PHB with 100% methanol and 35% H_2_O_2_. After 4 washings in 0.1 M PHB, sections were incubated in 0.1 M PHB containing 0.2% triton for 20 min and afterwards treated with 2 M HCl in a heated water bath (37 °C) for 30 min. Sections were washed with borate buffer (pH = 8.5) twice for 15 min each and kept in blocking solution consisting of 5% normal goat serum (NGS) diluted in 0.1 M PHB for 2 h. Then incubation with primary antibodies directed against BrdU or TH (Supplementary Table S1) followed overnight at 4 °C. Thereafter, sections were washed five times for 5 min each in 0.1 M PHB and incubated with secondary biotinylated antibodies (Supplementary Table S2) for 2 h at room temperature (RT), followed by 5 washing steps in 0.1 M PHB. After that, sections were incubated in the avidin-biotin complex solution (ABC-Kit, Vectastain) diluted with 0.1 M PHB. After 5 more washing steps, peroxidase activity was visualized by adding the chromogenic DAB solution (Sigma, DAB in 0.1 M PHB with 0.03% H_2_O_2_). After 1–2 min reaction became visible. Finally, the sections were mounted on glass slides and dehydrated in rising concentrations of ethanol, followed by 2 consecutive incubations in xylene for 5 min each before being covered.

### Immunofluorescence

Free-floating sections were incubated in 2 M HCl at 37 °C for 30 min and subsequently washed twice for 15 min in 0.1 M borate buffer (pH = 8.5). Afterwards, sections were washed 3 times in 0.1 M PHB and blocked with 5% NGS and 0.3% triton in 0.1 M PHB for 2 h at RT. Sections were then incubated with primary antibodies (Supplementary Table S1) diluted in PHB with 5% NGS overnight at 4 °C followed by 5-times washing in 0.1 M PHB for 5 min each and subsequent incubation with secondary goat antibodies (Supplementary Table S2) for 2 h at RT before being washed again in 0.1 M PHB 5 times for 5 min. These steps were conducted for each antibody sequentially to obtain a double or triple staining.

We performed the following triple staining procedures: We stained the OB for BrdU, NeuN and TH. The RMS behind the PB was stained for BrdU with Nestin and PSA-NCAM. For the detection of PSA-NCAM, we used a secondary biotinylated antibody against mouse IgM and streptavidine Cy5. Nestin was used first in row to avoid cross reactions. We stained the same area for BrdU, Nestin and GFAP and BrdU and Nestin with pax6. Sections were mounted in a 0.9% saline pool onto gelatin-coated slides and covered after 3–5 min in DABCO (1,4-diazabicyclo[2.2.2]octane) anti-fade mounting medium.

### TUNEL staining

To detect apoptotic cells in the OB, we used the Click-iT TUNEL Alexa Fluor 488 Assay (Invitrogen). Sections were incubated for 20 min in PHB + 0.3% Triton X-100. Mounted sections were heated twice in citrate buffer (pH = 5.8) until boiling and left in the hot citrate buffer for 20 min while cooling down. Afterwards, sections were blocked in 0.1 M PHB containing 5% NGS and 1:1000 diluted bovine serum albumin (BSA) for 1 h before TUNEL staining was finally performed, following the instructions of the kit. Sections were incubated in terminal deoxynucleotidyl transferase (TdT) reaction cocktail over night at 4 °C to avoid background staining. The following day, after another blocking step with NGS and BSA diluted in PHB and several washing steps, the Click-iT reaction was performed detecting modified dUTPs with fluorochrome Alexa Fluor 488. Subsequently, we stained the OB with TH.

### Microscopy

All microscopic analyses were conducted blinded concerning the identity of the animals. We performed confocal laser scanning microscopy with a TCS SP5 microscope (Leica, Wetzlar, Germany) and 3D analysis software, allowing unequivocal co-localization of BrdU and other differentiation markers. We examined the triple stainings BrdU/NeuN/TH, BrdU/Nestin/PAX6-GFAP and BrdU/Nestin/PSA-NCAM with orthogonal reconstructions of sections scanned with 1 µm thickness. For some cells we also performed the 3D reconstruction. To analyze the BrdU/NeuN/TH triple staining, we counted 50 BrdU^+^ cells and investigated their coexpression of NeuN and TH with 63x magnification. Confocal microscopy was also used to quantify apoptotic cells in the PGL labeled by TUNEL staining. TUNEL^+^ cells were counted by selecting 4 equal regions of the periglomerular zone in every fifth section of the OB with a magnification of 20×. Coexpression of BrdU/Nestin and other differentiation markers were qualitatively evaluated using orthogonal reconstruction of triple-labeled cells in order to verify colocalization in the x-y, x-z and y-z planes.

Cell counts were conducted on regularly spaced sections by using a semiautomatic stereology system (Stereoinvestigator 8.10, MicroBrightField, Magdeburg, Germany). We counted BrdU^+^ cells in the PGL and the GCL of the OB (3.7 mm to −6.9 mm relative to the anterior commissure), also in the SVZ, defined as the lateral wall of the lateral ventricle^31^ and in the RMS rostral of the PB (implanted +1.5 mm relative to bregma) until the beginning of the OB (+2.1 mm relative to bregma). We quantified the total number of TH^+^ cells in the PGL of the OB at all times of the experiment. For defining anatomical structures, a mouse brain atlas was used^30^. A systematic random procedure with optical fractionator and a counting frame size of 100 × 100, which was spaced in a 300 × 300 counting grid for the SVZ and OB and 100 × 100 counting grid for the RMS, was used for quantification.

### Statistics

Statistical analyses were done with Prism 7.03 (GraphPad Software, La Jolla, CA). Data were compared by two-way ANOVA with Tukey’s or Fisher’s LSD *post hoc* tests. Data are shown as mean + SEM. A p-value of <0.05 was considered as statistically significant.

## Supplementary information

Supplementary

## Data Availability

The data that support the findings of this study are available from the corresponding author.
