# Supplementary material for: Neuronal precursor cells with dopaminergic commitment in the rostral migratory stream of the mouse
Source: Sci Rep. 2019 Sep 16;9:13359. doi: 10.1038/s41598-019-49920-5 (PMC6746949; doi:10.1038/s41598-019-49920-5)
Supplement: Supplementary file 1 — Supplementary [file 41598_2019_49920_MOESM1_ESM.docx]

**Neuronal precursor cells with dopaminergic commitment in the rostral migratory stream of the mouse**

**Kerstin Schweyer, Corinna Rüschoff-Steiner, Oscar Arias-Carrión, Wolfgang H. Oertel, Thomas W. Rösler & Günter U. Höglinger**

**Supplementary Table S1:** Primary antibodies.

| Antigen | Species | Concentration | Source | Cat. No. |
| --- | --- | --- | --- | --- |
| GFAP | Rabbit | 1:200 | Thermo Fisher Scientific | 18-0063 |
| NeuN | Mouse | 1:1000 | Merck-Millipore | MAB377 |
| BrdU | Rat | 1:500 | AbD Serotec | OBT003 |
| Nestin | Mouse | 1:300 | Becton-Dickinson Biosciences | 556309 |
| PSA-NCAM | Mouse | 1:500 | Merck-Millipore | MAB5324 |
| TH | Rabbit | 1:1000 | Merck-Millipore | AB152 |
| Pax6 | Rabbit | 1:200 | Abcam | ab5790 |
| Click-iT® TUNEL |  |  | Invitrogen | C10245 |

**Supplementary Table S2.** Secondary antibodies.

| Antibody | Host | Concentration | Source | Cat. No. |
| --- | --- | --- | --- | --- |
| Biotin-SP (long spacer) AffiniPure IgG (H+L)  Anti-Rat | Donkey | 1:500 | Jackson Immunologicals | 712-065-153 |
| Cy™3-AffiniPure F(ab')2 Fragment IgG (H+L)  Anti-mouse | Goat | 1:500 | Jackson Immunologicals | 115-166-003 |
| Cy™3 AffiniPure IgG (H+L)  Anti-rabbit | Goat | 1:500 | Jackson Immunologicals | 111-165-003 |
| Alexa Fluor 488  IgG (H+L) Anti-rat | Goat | 1:1000 | Thermo Fisher Scientific | A11006 |
| IgM Biotin conjugated Anti-mouse | Goat | 1:500 | Sigma-Aldrich | B 9265 |
| Cy™5 Streptavidin-conjugated | Goat | 1:500 | Thermo Fisher Scientific | AB152 |
| Cy™5 AffiniPure IgG (H+L)  Anti-rabbit | Goat | 1:500 | Jackson Immunologicals | 111-175-144 |
